# Current and Evolving Biomarkers in the Diagnosis and Management of Testicular Germ Cell Tumors

**DOI:** 10.3390/jcm13237448

**Published:** 2024-12-06

**Authors:** Jennifer Sykes, Alain Kaldany, Thomas L. Jang

**Affiliations:** 1 Division of Urology, Rutgers Robert Wood Johnson Medical School, New Brunswick, NJ 08901, USA; js3545@rwjms.rutgers.edu (J.S.); alainkaldany@gmail.com (A.K.); 2 Division of Urologic Oncology, Rutgers Cancer Institute of New Jersey, New Brunswick, NJ 08901, USA

**Keywords:** biomarkers, serum tumor markers, testicular cancer, germ cell tumor, microRNA

## Abstract

Testicular cancer is the most common cancer among young adult men and has favorable outcomes, with survival rates approaching 99% and over 80% for those with early and advanced stage disease, respectively. Biomarkers play a critical role in the diagnosis, pre-treatment risk stratification, surveillance, and assessment of post-treatment disease response in these men. Traditional serum tumor markers (STMs), which include alpha fetoprotein (AFP), beta subunit of human chorionic gonadotropin (β-hCG), and lactate dehydrogenase (LDH), are limited by low sensitivity (approximately 50%) during initial diagnosis; false-positive elevations as a result of other benign and malignant conditions; and negative levels in low-stage disease and in certain histologies such as teratoma and seminoma. As a result, novel biomarkers with potentially better performance characteristics, including microRNA (miRNA), circulating tumor DNA (ctDNA), and circulating tumor cells (CTCs), are being investigated. MicroRNAs are small noncoding RNA involved in transcription and translation and regulate the expression of almost one-third of human genes that regulate the cell cycle, differentiation, proliferation, and apoptosis. In germ cell tumor (GCT) patients, miR371a-3p has been identified as a promising biomarker with sensitivity and specificity of approximately 90–92% and 84–86%, respectively. The use of this new biomarker could aid in several clinical scenarios, such as predicting the presence of micrometastases in chemotherapy-naïve patients with clinical stage I–II disease, thereby guiding decisions on treatment versus surveillance and predicting the presence of viable GCT in patients with residual disease post chemotherapy. Clinical trials are ongoing to validate the use of miRNA 371 as a biomarker and to define its performance characteristics. Though promising, miRNAs are limited by their inability to detect teratoma. ctDNA and CTCs are two other emerging biomarkers, though further studies are needed to clarify their role in managing patients with GCT.

## 1. Introduction

Testicular cancer is the most commonly diagnosed tumor in young adult men aged 20–39 years [1]. The annual incidence in the United States (US) is 5.7 per 100,000 adult men, with an estimated 9760 new US cases in 2024 [1]. Testicular cancer, termed testicular germ cell tumors (TGCT), most commonly arises from the cells of sexual reproduction, and comprises roughly 95% of all testicular neoplasms [2]. TGCT are further categorized into seminoma and nonseminomatous germ cell tumors (NSGCT). Mainstays of treatment, which are contingent on histology, stage, and risk, include radical inguinal orchiectomy (or rarely testis-sparing surgery) followed by surveillance, chemotherapy, radiotherapy, and/or retroperitoneal lymph node dissection. The prognosis for men with TGCT is overall favorable, with long-term survival for men with early stage disease approaching 99%, and for those with advanced-stage disease exceeding 80% [3]. As such, management strategies have focused on reducing morbidity and the potential harms of treatment and minimizing overtreatment in this young population with a highly curable disease.

Biomarkers play a critical role in the diagnosis, pre-treatment risk stratification, surveillance, and assessment of post-treatment disease response in these men. Traditional serum tumor markers (STMs) include alpha fetoprotein (AFP), beta subunit of human chorionic gonadotropin (β-hCG), and lactate dehydrogenase (LDH). However, these classic STMs are limited by overall low sensitivity (approximately 50%), negative levels in those with early stage disease or histologies that do not typically overexpress these markers, such as teratoma and seminoma. Finally, false-positive elevations as a result of other benign and malignant conditions can be seen [4].

STM levels at the time of diagnosis are prognostic and have been incorporated into the AJCC Staging System for Testicular Cancer (TNMS Staging System). Typically, TNM is used for cancer staging, but testicular cancer is unique in that it includes serum tumor markers in the staging process. The S stage is characterized by the degree of elevation of the serum tumor markers, separated into Sx, S0, S1, S2, and S3 [5]. Furthermore, for patients with advanced disease, STMs have been integrated into the International Germ Cell Cancer Collaborative Group (IGCCCG) risk classification with other clinical parameters, to assess prognosis and dictate the chemotherapy regimen. The risk stratification is divided into good-, intermediate-, and poor-risk strata [6,7].

The sensitivity of AFP, β-hCG, LDH alone, and in combination is relatively low. Among all men with testicular GCT (NSGCT and seminoma), Dieckmann and investigators found in their study that the sensitivity of AFP was 18%, β-hCG 35%, LDH 28%, and about 50% for all three STMs combined. For NSGCT patients, the sensitivities of AFP, β-hCG and LDH were 44%, 40%, and 29%, respectively, and 58% for all 3 STMs combined. For seminoma patients, the sensitivities of AFP, β-hCG, and LDH were 2.3%, 31%, and 28%, respectively, and 46% for all 3 STMs combined [8]. Finally, traditional STMs are nonspecific for testicular GCTs, with each biomarker having elevations in other physiologic and pathologic states. Given the low sensitivity and specificity of these classic STMs, as well as the importance of accurate monitoring in this patient population, research investigations have focused on developing novel biomarkers with improved accuracy and performance characteristics. In this review, we present an overview of traditional STMs in TGCT and discuss several novel biomarkers including microRNA, circulating tumor DNA (ctDNA), and circulating tumor cells (CTCs).

## 2. Discussion

### 2.1. Traditional Serum Tumor Markers for Testicular Germ Cell Tumors

#### 2.1.1. Alpha Fetoprotein (AFP)

AFP is a serum binding protein produced by the fetal yolk sac, liver, and gastrointestinal tract. Its highest concentration is 3 mg/mL at 12–14 weeks gestation, and subsequently decreases to <40 ng/mL 1 year after birth [9]. AFP has a half-life of about 5–7 days. Post-orchiectomy, AFP levels are anticipated to normalize in most patients by 4 to 5 half-lives or approximately one month, in the absence of persistent or metastatic disease [10]. In terms of its utility in germ cell tumors, AFP is generally elevated in embryonal cell carcinoma as well as yolk sac tumors but is not elevated in pure choriocarcinoma or pure seminoma. Though teratoma traditionally does not produce elevations in STMs, mild AFP elevations can be observed in up to 25% of teratomas owing potentially to hepatoid differentiation or mucinous glandular components [11]. AFP is elevated in about 50–70% of those with low-stage NSGCT and 60–80% of those with advanced-stage NSGCT. Several other conditions can result in AFP elevation, including hepatocellular carcinoma, pancreatic cancer, gastric cancer, colorectal cancer, and bronchial cancer [12]. Other benign causes of AFP elevation include viral hepatitis, cirrhosis, and liver trauma. The sensitivity of AFP alone for detecting TGCT is poor and has been reported as 13% to 18% [8,13].

#### 2.1.2. Human Chorionic Gonadotropin (hCG)

hCG is produced during pregnancy by syncytiotrophoblast cells of the placenta, maintaining the corpus luteum. In germ cell tumors, hCG is produced either as an intact molecule comprising alpha and beta subunits or the beta subunit alone. All patients with choriocarcinoma produce hCG, while 40–60% of patients with embryonal cell carcinoma have elevated hCG. In 10–20% of patients with pure seminoma, generally mild elevations of β-hCG levels can be observed [10]. The half-life of β-hCG is 24–36 h [14]. As hCG is composed of an alpha and beta subunit, there may be cross-reactivity with the alpha subunit of LH which can cause a false positive. In some cases, hypogonadism induces LH and hCG production by the pituitary gland causing elevation of hCG. Administration of testosterone should correct the false elevation of hCG within 48 to 72 h. A variety of tumors also produce hCG including liver, biliary, pancreas, stomach, breast, kidney, and bladder cancer. The sensitivity of hCG alone for detecting TGCT has been reported at 34.5% to 38% [8,13].

#### 2.1.3. Lactate Dehydrogenase (LDH)

Of all the classic STMs, LDH is the least specific for GCT. There are 5 isoforms of LDH and LDH isoform 1 (LDH1) is the biomarker utilized for TGCT. LDH catalyzes the conversion between lactate and pyruvate and is released into the blood during cell death, thus making it nonspecific to GCTs. The half-life of LDH is around 4–5 days [4]. The highest elevation of LDH is observed in patients with advanced-stage disease and its interpretation has been indicative of high cellular turnover and disease burden. The sensitivity of LDH alone for detecting TGCT has been reported at 28% [4,8].

#### 2.1.4. Accuracy of Traditional Serum Tumor Markers

Dieckmann et al. studied the classic serum tumor markers and made critical contributions to how we view their utility. The authors showed that AFP, β-hCG, and LDH are elevated less than 50% of the time in patients with GCT, and elevation in any single marker occurs in less than 60% of the patients; in other words, the combined sensitivity of all traditional STMs is roughly 50% [8]. The pattern of marker elevation was shown to depend on histological subtype, clinical stage, pathological stage, tumor size, and age. More specifically, these investigators reported that among all GCT patients (seminoma and NSGCT), the sensitivity of AFP was 18%, β-hCG 35%, LDH 28%, and about 50% for all three STMs combined among GCT. For NSGCT patients, the sensitivities of AFP, β-hCG, and LDH was 44%, 40%, and 29%, respectively, and 58% for all 3 STMs combined. For seminoma patients, the sensitivities of AFP, β-hCG and LDH was 2.3%, 31%, and 28%, respectively, and 46% for all 3 STMs combined. The association between biomarker levels and treatment response was confirmed; however, LDH was shown to remain elevated in 30% of patients despite cure. Lastly, the authors showed that with relapse, close to 50% of patients had elevated tumor markers, but the pattern of elevation had changed in almost 50% of these patients [15]. This research highlighted the need for more sensitive and specific biomarkers in this disease.

### 2.2. Clinical Utility of Novel Biomarkers

There are numerous areas within TGCT management that would benefit significantly from more sensitive and specific biomarkers to help guide management:

#### 2.2.1. Identifying Seminoma

There is no biomarker specific to testicular seminoma; only up to 20% of patients with seminoma have elevated β-hCG, and elevated β-hCG is not specific to seminoma as it may be elevated in other malignancies [15].

#### 2.2.2. Clinical Stage I (CSI) Seminoma

Nearly 80% of patients with seminoma present with CSI disease at the time of diagnosis. Stage I seminomas are managed initially with radical orchiectomy and the risk of recurrence is generally 15% to 20%, often within the first year, as a result of subclinical metastasis at the time of presentation [16]. The size of the primary testicular tumor is associated with the risk of relapse. Tumor size appears to be a continuous variable, with larger tumor sizes conferring a greater risk of relapse. However, even for tumors greater than 7 cm, the risk of relapse is less than 30% [17,18]. The presence of rete testis invasion in the orchiectomy specimen has been found in some studies to be associated with a higher risk of relapse but has not been validated in other investigations [19,20].

Options for managing CSI seminomas include surveillance, chemotherapy, or radiation. As most patients are cured by orchiectomy alone, surveillance is the preferred post-orchiectomy management for patients. A retrospective study including 1344 patients with CSI seminoma on surveillance demonstrated a 5-year survival of 99%, highlighting the appropriate role of surveillance in this patient population [21]. For patients who are unable to comply with the surveillance schedule, chemotherapy with 1–2 doses of single-agent carboplatin or radiation therapy are alternate options. A prospective study comparing treatment outcomes of patients with CSI seminoma managed with surveillance, one cycle of carboplatin, or 2 cycles of carboplatin showed that crude relapse rates were higher with the one-cycle regimen (5%) compared to the two-cycle regimen (1.5%). The relapse-rate for the surveillance group in this study was 8.2% [22]. A study comparing outcomes of one cycle of chemotherapy and radiation therapy showed comparable relapse free and survival-free rates at about 95% [23]. Chemotherapy carries risks of cardiac toxicity and secondary malignancies, while radiation is associated with an 80% increase in the risk of death from a secondary malignancy. As all three management options result in an overall survival near 100%, surveillance remains the preferred management strategy.

#### 2.2.3. Clinical Stage I NSGCT

The risk of relapse for patients with Stage I NSGCT post orchiectomy ranges from 15% to 50% [24]. The presence of lymphovascular invasion (LVI) in the primary testicular tumor is a known risk factor for patients with Stage I NSGCT that increases the risk of relapse to nearly 50% [25].

Options post orchiectomy for this group of patients include surveillance, adjuvant chemotherapy with one cycle of bleomycin, etoposide, cisplatin (BEP), or nerve-sparing retroperitoneal lymph node dissection (RPLND). Surveillance is considered the preferred approach in this group of patients, particularly those without LVI in the orchiectomy specimen, given the potential long-term toxicities associated with adjuvant treatments. Long-term toxicities associated with chemotherapy include risk of secondary malignancies, ototoxicity, nephrotoxicity, cardiovascular effects, and infertility, which is especially relevant in this young population that testicular cancer afflicts [26]. RPLND, though a highly effective treatment in this population with few long-term adverse effects, requires an invasive procedure with the possibility of retrograde ejaculation. With contemporary nerve-sparing RPLND approaches, antegrade ejaculation can be preserved in the majority of patients. Treating all patients with CSI disease with adjuvant therapy would expose 70% of patients who never would have recurred to unnecessary treatment [27]. A sensitive and specific biomarker would better risk-stratify patients to identify which of these patients are at the highest risk of recurrence and which patients would benefit from adjuvant therapy. It would also limit the number of cumulative tests and imaging studies patients on surveillance would need to undergo.

#### 2.2.4. Stage IIA GCT with Negative Markers

For patients with a retroperitoneal lymph node mass <2.0 cm and the absence of elevated STMs, there is uncertainty as to whether these lesions represent disease versus inflammatory or infectious processes. In contemporary surgical series, the risk of misdiagnosis can be significant, with 40% to 50% of patients harboring no tumor at the time of surgery [28]. Therefore, in this clinical scenario, there remains a risk of misdiagnosis and either over- or undertreatment of disease.

#### 2.2.5. Assessment of Residual Disease in the Post-Chemotherapy Setting for Advanced GCT

Typically, patients with advanced NSGCT are treated with chemotherapy followed by RPLND for residual disease in excess of 1 cm to remove any teratoma (40%) and any residual disease (10–15%) [29]. This means that almost 40% to 50% of patients who undergo RPLND in the post-chemotherapy setting undergo surgery unnecessarily [30].

In patients with seminoma, PET-CT scan imaging has been shown to have a high negative predictive value in masses greater than 3 cm in the post-chemotherapy setting. However, positive predictive values remain low because of high rates of false-positive results associated with desmoplastic reaction of seminomas to chemotherapy [31,32].

### 2.3. Novel Biomarkers in Testicular Germ Cell Tumors

#### 2.3.1. MicroRNA

The most promising novel biomarker for TGCT is micro ribonucleic acids (miRNA). MicroRNA are single-stranded, non-coding RNA consisting of 20–23 nucleotides that work by binding to messenger RNA and are present at detectable levels in serum. Since their discovery, there are few miRNAs with known function. Their functions are generally deduced by their activity on a target gene. Generally, target genes with sequences that are completely complementary to the miRNA will be degraded, while targets with partially complementary sequences will have their translation inhibited in addition to some mRNA degradation [33]. A single gene may be targeted by multiple miRNAs, and a single miRNA may target multiple genes [34]. miRNA have been found to affect angiogenesis, tumor invasion, energy metabolism, and immune system activation, implicating them as contributors to oncogenesis [35]. miRNA expression in cancer appears to be tissue-specific and have short half-lives, making them good biomarkers to use for disease monitoring/response and recurrence [36]. These molecules remain stable after their release from cells and can be measured by quantitative polymerase chain reaction (qPCR) [37]. Table 1 outlines the studies identifying miRNA as viable tumor markers for GCTs.

In 2006, miRNA-372 and -373 were identified as oncogenes directly targeting large tumor suppressor homolog 2 (LATS2) in germ cell tissue and cell lines [38]. miRNA 371-373 have been shown to be highly overexpressed in all malignant GCTs (aside from pure teratomas) regardless of patient age, histology, or anatomic site of the tumor [39]. Patients without GCTs did not express the marker, making miR-371a-3p biologically specific for GCT. miR-371a-3p has achieved sensitivities of 84.7%, 88.7%, and 89% along with specificities of 99%, 93.4%, and 90%, far surpassing the sensitivity and specificity of the current STMs which have a combined sensitivity of only 50% [8,13,40]. In a prospective study, Nappi et al. found that miR371 expression in confirmed GCT had specificity, sensitivity, PPV, and NPV of 100%, 96%, 100%, and 98%, respectively [41]. miRNA concentration has been shown to positively correlate with disease burden; patients with more advanced disease had higher levels of miRNA 371a-3p. miRNA levels also correlated with treatment effects; miR-371a-3p levels decreased in patients who completed chemotherapy [8].

In patients who are post radical orchiectomy, a decrease in miR-371a-3p was reported in 91.77% of patients with local disease and 82.4% of patients with metastatic disease [42]. There was persistent expression in a small proportion of clinical stage I patients which could be related to occult metastasis. Thus, miRNA concentration could signal the presence of viable tumor cells after orchiectomy. After chemotherapy, miRNA levels remained elevated in patients with viable germ cell tumor in post-chemotherapy RPLND specimens [43].

In a prospective study evaluating patients undergoing RPLND, miR-371a-3p expression was 13,000 times higher than in teratoma or benign tissue [44]. Other studies showed similar findings of significantly higher pre-RPLND levels of miRNA 371-a-3p, with a decrease following the procedure. In a prospective study, 94.1% of patients with disease relapse experienced higher levels of miR-371a-3p than post-orchiectomy levels, while only 34.1% of these patients had an increase in STMs [45]. Thus, this marker allows for earlier relapse detection and a shorter time to treatment.

Though miR371 holds promise as a sensitive and specific biomarker for nonteratomatous GCT, miR371 is not a reliable biomarker to detect teratoma. miR375 has been shown to be elevated in teratomas; however, its use as a single biomarker is not reliable. Nappi et al. showed that the combined use of miR375 and miR371 improved the accuracy of detecting teratoma with an AUC of 0.77 (95% CI: 0.62–0.93) as opposed to miRNA375 alone (AUC 0.55, 95% CI: 0.36–0.74) [46]. Their study showed that patients with teratoma expressed high levels of miR375 and undetectable levels of miR371, while the presence of miRNA371 indicates nonteratoma histology.

**Table 1:** Summary of studies looking at miR371 as a biomarker for testicular germ cell tumor. PPV = positive predictive value, NPV = negative predictive value, AUC = area under the curve.

Study	Cohort	Sensitivity	Specificity	PPV	NPV	AUC
Dieckmann et al. [8] 2017 2019 [15]	All GCT 616 men with GCT 258 controls	88.7%	93.4%	97.2%		0.94
		90.1%	94%	97.2%		0.966
Nappi et al. [41] 2019	All GCT 111 men with GCT	96%	100%	100%	98%	
Syring et al. [13] 2015	All GCT 30 men with GCT 18 controls	84.7%	99%			
Lafin et al. [44] 2020	Prior to RPLND, chemotherapy-naïve patients 24 men with GCT	100%	92%			0.965
Leao et al. [43] 2018	Post chemotherapy, pre RPLND 82 men with GCT	100%	54%			0.874
Van Agthoven et al. [40] 2017	All GCT 250 men with GCT	90%	86%	94%	79%	0.951
Badia et al. [47] 2021	All GCT Pre orchiectomy 69 men, 58 with GCT	93%	100%	100%	73%	0.978

#### 2.3.2. Circulating Tumor DNA (ctDNA)

Circulating tumor DNA cells are tumor-specific fragments of DNA that are detectable in body fluids such as blood [48]. These are released into the blood during cell death. ctDNA has evolved as a biomarker for various malignancies and is in its early stages of exploration as a potential biomarker for TGCT. ctDNA plays an important role particularly for tumors that are too small to appear on radiographic imaging. The first generation of ctDNA was not ideally suited as a tumor marker for this low-volume state. However, a newer, more sensitive and specific ctDNA assay has been developed for solid tumors called molecular residual disease (MRD) tests that can detect micrometastasis. Some tests (signatera and personalized cancer monitoring) use whole exome sequencing, while others (precise MRD) use whole genome sequencing of the tumor to develop a personalized mutation profile [49]. An early study showed that patients with TGCT (both seminoma and nonseminoma) had a higher level of ctDNA compared with the control group with a sensitivity of 88% and a specificity of 97%. Levels of ctDNA corresponded to disease stage and activity, and this study included men who had normal STMs [50]. One study of 35 patients showed pre-orchiectomy ctDNA in 91.6% of patients with stage I disease and 100% of stage II or III patients [51]. Another study of 25 patients demonstrated a 0% recurrence rate in patients who were ctDNA-negative after undergoing chemotherapy or surgery [52]. Both of these studies used the tumor-informed commercially available Signatera assay. These preliminary studies have shown that the sensitivity and specificity of ctDNA are higher than traditional serum tumor markers, although it appears outperformed by miRNA [53]. Though these data suggest ctDNA may potentially improve the clinical management of patients with TGCT, further studies will be required to determine its utility as a biomarker in this disease.

#### 2.3.3. Circulating Tumor Cells (CTCs)

Circulating tumor cells may be found in the serum of patients with solid tumors and have the potential to seed metastasis. One study found CTCs in 11.5% to 17.5% of patients with GCTs; however, a higher proportion of patients with metastatic cancer had detectable CTCs [54]. The challenge with CTCs is in their identification. A variety of protein markers have been studied to identify CTCs but have shown an overall low sensitivity of less than 60% [55]. Furthermore, when detected, CTCs have not always been associated with tumor burden. These issues, along with challenges in the detection and isolation of CTCs, limit their clinical utility as a GCT biomarker.

#### 2.3.4. Future Direction

Research on the use of miRNA as a biomarker for GCTs is still in its infancy. The literature has shown variability in the use of serum versus plasma miRNA for analysis, and there is no consensus on the cut-off or threshold of normal miRNA levels. Further trials are needed to standardize these variables before miRNA can be used in the clinical setting.

Most importantly, further research is required to isolate a more sensitive and specific biomarker for teratoma. Despite promising studies combining miR371 and miR375 to determine the presence of teratoma, there are questions about the reproducibility of these findings across different cohorts and clinical settings. Until a more validated biomarker is found for teratoma, this limits the use of miR371 as a biomarker in the setting of post-chemotherapy residual masses, as up to 40% of these patients may harbor teratomas [4].

There are currently several clinical trials underway to further validate the utility of miR 371a-3p. The trials listed were found through a thorough search of www.clinicaltrials.gov (accessed on 24 July 2024) and are listed below (Table 2):

SWOG-S1823 studies whether miR371 can predict the chance of cancer returning in patients with GCTs. In this study, patients undergo blood collection every 3–6 months for up to 3 years [56].

AGCT1531; NCT03067181 is a phase III trial showing how well active surveillance helps physicians monitor patients with low-risk GCTs for recurrence. The trial also studies whether carboplatin or cisplatin is the preferred chemotherapy modality in treating metastatic standard-risk GCTs. Throughout this study, serum miRNA in stage I testicular cancer patients will be collected [57].

The MAGESTIC trial (NCT06060873) is a phase II trial studying the accuracy of miR 371 in predicting preoperative active germ cell malignancy.

NCT04914026 is a cohort study that will assess the sensitivity and specificity of miR 371 in detecting TGCT. This trial will collect blood samples from patients and measure miR371 at the time of orchiectomy, during treatment, in the surveillance period, and in early detection of recurrence.

EMIT (NCT03980587) is a retrospective study that will look at whether ctDNA is detectable in the plasma of patients with platinum-refractory GCT, and then analyze the molecular aberrations.

Data from these trials will be critical in validating these new biomarkers for clinical use.

## 3. Conclusions

Management of testicular germ cell tumor has long been guided by serum tumor markers including AFP, β-hCG, and LDH. However, their clinical utility is limited by poor sensitivity and specificity. Emerging biomarkers, including miRNA, circulating tumor DNA (ctDNA), and circulating tumor cells (CTCs), have demonstrated potential to improve the management of testicular cancer across various clinical settings such as diagnosis and staging, prognostication, disease monitoring, and surveillance. Still, research on these novel biomarkers is in its early phases, and more data regarding performance characteristics will be required to prove their utility for widespread clinical use in patients with testicular cancer. Forthcoming clinical trials, such as MAGESTIC and SWOG-S1823, will be critical in validating these biomarkers for generalized use.

## Figures and Tables

**Table 2:** Ongoing and upcoming prospective clinical trials assessing novel biomarkers in testicular germ cell tumor. NSGCT = nonseminomatous germ cell tumor, PPV = positive predictive value, EFS = event-free survival, ctDNA = circulating tumor DNA.

Trial Name	CT Identifier	Study Type/Phase	Status	Biomarker	Target Enrollment	Population	Outcome of Interest
A Prospective Observational Cohort Study to Assess miRNA 371 for Outcome Prediction in Patients With Newly Diagnosed Germ Cell Tumors (SWOG-S1823)	NCT04435756	Observational	Recruiting	miR-371	956	Seminoma and NSGCT	PPV of miR-371
A Phase 3 Study of Active Surveillance for Low Risk and a Randomized Trial of Carboplatin vs. Cisplatin for Standard Risk Pediatric and Adult Patients With Germ Cell Tumors	NCT03067181	Interventional, Phase III	Recruiting	miRNA	2097	Stage I Seminoma and NSGCT	EFS via tumor marker decline
MiRNA in Detecting Active Germ Cell Tumors in Early Suspected and MetastaTIC Disease Trial (MAGESTIC)	NCT06060873	Observational, Phase II	Recruiting	miR-371	418	Stage I or IIA/B Seminoma and NSGCT undergoing RPLND	PPV of miR-371
MicroRNA-371 as Markers for Disease Activity and as a Tool to Monitor the Effect of Chemotherapy and Early Detection of Recurrence in Patients With Testicular Germ Cell Tumours	NCT04914026	Observational	Recruiting	miR-371	350	Seminoma and NSGCT, all stages	Sensitivity and specificity of miR-371
Exploratory Study of Molecular Characterization in Patients With Metastatic Germ Cell Tumours Refractory/Resistant to Platinum Treatment	NCT03980587	Observational	Not yet recruiting	ctDNA	18	Metastatic platinum-resistant GCT	Measurable ctDNA
